# Co-expression of CD40L with CD70 or OX40L increases B-cell viability and antitumor efficacy

**DOI:** 10.18632/oncotarget.10068

**Published:** 2016-06-15

**Authors:** Chang-Ae Shin, Hyun-Woo Cho, A-Ri Shin, Hyun-Jung Sohn, Hyun-Il Cho, Tai-Gyu Kim

**Affiliations:** ^1^ Department of Microbiology and Immunology, College of Medicine, The Catholic University of Korea, Seoul 137-701, South Korea; ^2^ Catholic Hematopoietic Stem Cell Bank, College of Medicine, The Catholic University of Korea, Seoul 137-701, South Korea; ^3^ Catholic Cancer Research Institute, College of Medicine, The Catholic University of Korea, Seoul 137-701, South Korea

**Keywords:** cancer vaccine, B-cells, costimulatory ligand, anti-apoptosis, tumor immunity

## Abstract

Activated B-cells are a promising alternative source of antigen-presenting cells. They can generally be obtained in sufficient numbers for clinical use, but in most instances produce weak immune responses and therapeutic effects that are suboptimal for use in therapeutic cancer vaccines. To improve the immunogenic potency and therapeutic efficacy of B-cell-based vaccines, *ex vivo*-activated B-cells were transduced with recombinant lentiviruses in order to express additional costimulatory ligands—CD40L, CD70, OX40L, or 4-1BBL—either individually or in pairs (CD70/CD40L, OX40L/CD40L, or 4-1BBL/CD40L). We observed that the expression of CD40L molecules on B-cells was crucial for T-cell priming and activation. Administration of B-cells co-expressing CD40L with the other costimulatory ligands provided substantial antigen-specific CD8 T-cell responses capable of provoking *in vivo* proliferation and potent cytolytic activities. Notably, expression of CD40L augmented B-cell viability by inhibiting apoptosis through upregulated expression of the anti-apoptotic molecules BCL2, Bcl-xL and Bax. B-cells co-expressing CD40L with CD70, OX40L, or 4-1BBL induced potent therapeutic antitumor effects in a B16 melanoma model. Moreover, the combination of genetically-modified B-cell vaccines with programmed cell death-1 blockade potentiated the therapeutic efficacy. These results suggest that B-cells endowed with additional costimulatory ligands enable the design of effective vaccination strategies against cancer.

## INTRODUCTION

Since cytotoxic CD8 T-cells are key immune effectors that eradicate malignant cells, a prerequisite for cancer immunotherapy is to activate and expand tumor-reactive CD8 T-cells capable of recognizing and destroying tumor cells [1, 2]. The activation of CD8 T-cells requires additional costimulatory signals offered by antigen-presenting cells (APCs) such as dendritic cells (DCs), along with the engagement of T-cell receptors (TCRs) with cognate peptide-major histocompatibility complex (pMHC) class-I molecules [3]. Mature DCs, which express high levels of MHC molecules and costimulatory ligands, have been shown to be efficient cellular adjuvants that evoke and augment tumor-reactive CD8 T-cell responses both *in vitro* and *in vivo* [4, 5]. Moreover, DCs genetically modified to express immune-stimulatory molecules, such as costimulatory ligands and cytokines, have elicited enhanced T-cell responses *in vitro* and *in vivo* [6, 7]. Clinical trials have been performed for various tumor types using antigen-loaded DCs, which could provide a potent new option for current cancer immunotherapeutic strategies in cellular vaccines [8, 9]. Although DC-based cellular vaccines have been shown to be safe and apparently immunogenic in cancer patients, no significant protective immunity has been achieved. Significant drawbacks include the limitations in obtaining sufficient cells for clinical applications and difficulty in genetic modification for use as a cellular adjuvant [10].

For some time, we and others have attempted to identify reliable sources of autologous APCs as an alternative to DCs for immunotherapy. Activated γδ T-cells have been proposed as an alternative type of professional APCs exhibiting efficient antigen-presenting capabilities that stimulate naïve T-cell priming and proliferation [11]. CD4 T-cells have also been shown to evoke functional memory CD8 T-cell responses, and the expression of costimulatory CD80 and 4-1BBL on *in vitro*-expanded CD4 T-cells augments therapeutic antitumor immunity *in vivo* [12]. Likewise, numerous reports have shown that B-cells that are activated *in vitro* by treatment with inflammatory cytokines, CD40L, and Toll-like receptor (TLR) ligands, are promising alternative APCs for inducing efficient expansion of antigen-specific CD4 and CD8 T-cells and potentiating antitumor immunity *in vivo* [13–16]. In other reports, B-cells loaded with tumor antigens and the invariant natural killer T (NKT)-cell ligand α-galactosylceramide induced a wide range of adaptive immunity against tumor cells and activated NKT-cells [17, 18]. A previous report showed that genetically modified B-cells expressing the costimulatory molecules, OX40L and 4-1BBL, cytokine IL-12, and antigen synergistically augment CD8 T-cell proliferation as efficiently as DCs *in vitro* [19]. Furthermore, a recent study reported that B-cells are capable of efficiently cross-presenting tumor-specific antigens captured by tumor-derived autophagosomes, subsequently leading to effective antitumor immunity [20]. Nonetheless, a cellular vaccine using genetically modified B-cells that can enable the direct stimulation of naïve CD8 T-cells resembling mature DC functions in a tumor model has not been developed. Here, we test the hypothesis that *ex vivo*-activated B-cells modified to express CD40L may possess a superior ability to prime and expand antigen-specific T-cells due to the engagement of CD40 by CD40L on APCs in T-cell priming and activation.

## RESULTS

### Activated B-cells are efficiently transduced with lentiviral vectors encoding costimulatory molecules

**Figure 1:**
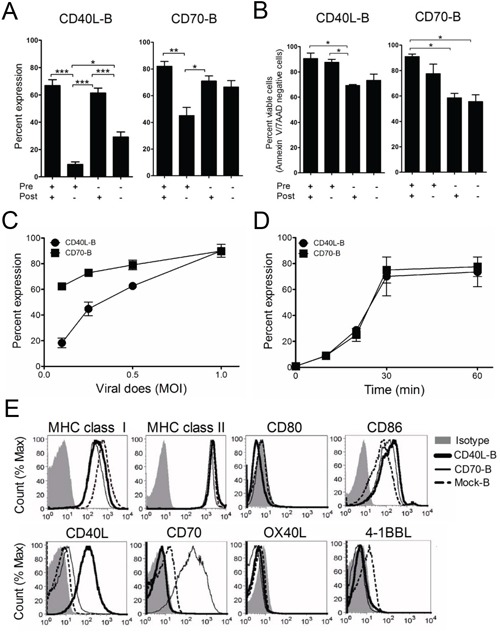
Expression of costimulatory molecules on *ex vivo*-activated B-cells Freshly isolated B-cells from spleen using immunomagnetic beads were pre-activated with (+) or without (−) anti-CD40 antibodies for 18 h. Then, the *ex vivo*-activated B-cells were transduced with recombinant lentiviruses encoding the costimulatory molecule CD40L or CD70 (CD40L-B and CD70-B), followed by co-culture with (+) or without (−) anti-CD40 Antibodies in the presence of IL-4. **A.** The expression of CD40L and CD70 in the transduced B-cells were examined by flow cytometry at 1 day post-transduction. Results represent the mean percentage from 2-independent experiments with SD (*bars*). **B.** Cell apoptosis of the transduced B-cells were assessed by staining with annexin-V and 7-AAD at 1 day post-culturing in the presence of IL-4. Results represent the mean percentage of annexin-V and 7-AAD-negative cells from 2-independent experiments with SD (*bars*). P values were calculated using 1-way ANOVA test (*P < 0.05; **P < 0.01; ***P < 0.001). **C.** Transduction efficacy of lentiviruses encoding CD40L and CD70, titrated according to various multiplicities of infection (MOI) from 0.1 to 1. **D.** Determination of optimal centrifugation time for transduction to *ex vivo*-activated B-cells. **E**. Investigation of surface expression of T-cell activating molecules. After a 1-day culture, CD40L- or CD70-transduced B-cells were harvested and analyzed for surface expression of MHC class I/II, CD80/86, CD40L, CD70, OX40L, and 4-1BBL by flow cytometry. Non-transduced B-cells (mock-B) were included as control.

First, we determined the optimal *in vitro* conditions for transducing B-cells with recombinant lentiviruses encoding the costimulatory molecules CD40L and CD70 (hereafter referred to as CD40L-B and CD70-B-cells, respectively). To verify the impact of CD40 activation, B-cells were incubated with or without anti-CD40 antibodies before lentiviral transduction, followed by culture for 2 days with or without anti-CD40 antibodies in the presence of IL-4. As shown in Figure 1A and 1B, CD40 activation in B-cells after lentiviral transduction was more crucial for efficient gene expression, while the pre-activation of B-cells with anti-CD40 antibodies augmented the levels of CD40L and CD70 expression and viability of the genetically modified B-cells *in vitro*. The lentiviral transduction efficacy in B-cells correlated with viral dose (Figure 1C) and centrifugation time (Figure 1D); polybrene had no effect on transduction efficacy (data not shown). Moreover, CD40-activated (and mock-transduced) B-cells had insignificant expression of costimulatory molecules CD80, CD40L, CD70, OX40L, and 4-1BBL, which was unaffected after lentiviral transduction (Figure 1E).

### B-cells expressing additional costimulatory ligands stimulate antigen-specific CD8 T-cells

**Figure 2:**
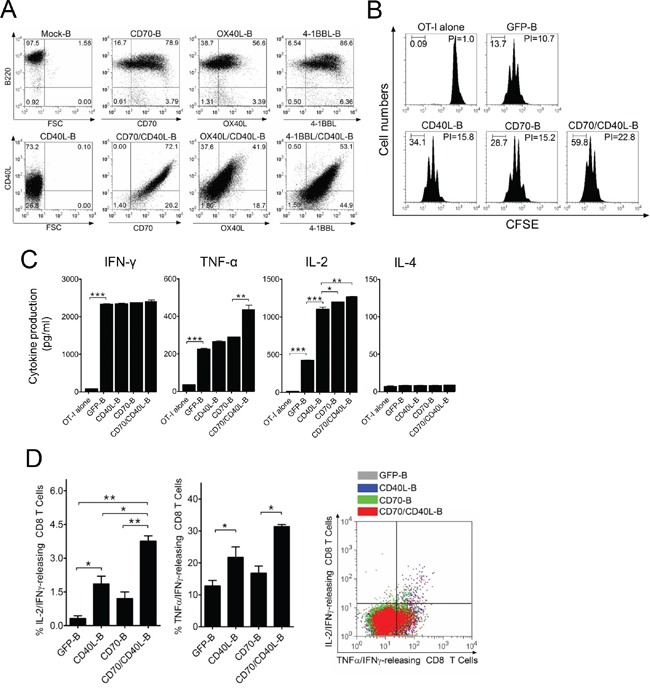
B-cells expressing additional costimulatory ligands stimulate antigen-specific CD8 T-cells *in vitro* **A.** *Ex vivo*-activated B-cells were transduced with recombinant lentiviruses encoding mouse CD40L, CD70, OX40L, or 4-1BBL either individually or in pairs (CD70/CD40L, OX40L/CD40L, or 4-1BBL/CD40L). After a 1-day culture, cells from each group were harvested and analyzed for surface expression of the transduced costimulatory molecules by flow cytometry. Non-transduced B-cells (mock-B) were included as control. **B.** Proliferation of OT-I cells in response to genetically modified B-cells. OT-I (CD45.2) cells were labeled with 5 μM CFSE and cultured with GFP-, CD40L-, CD70- or CD70/CD40L-expressing B-cells (GFP-B, CD40L-B, CD70-B, and CD70/CD40L-B, respectively), which were loaded with Ova_257_ peptide. On day 4 post-co-culture, cell proliferation was measured by flow cytometry and analyzed using Modfit LT software. Freshly CFSE-labeled cells were used as the parental cells, and their homogeneity was verified at the start of each experiment. A group of cultured OT-I cells alone was included as control (OT-I alone). The percentage of cells in each division obtained in a representative experiment is inserted in the graphs. *PI*, proliferation index: the sum of the cells in all generations divided by the computed number of parental cells present at the start of experiment. These experiments were repeated twice with similar results. **C.** Evaluation of cytokine profiles from OT-I cells co-cultured with variously conditioned B-cells for 4 days. The culture supernatants were measured for indicated cytokines using ELISA assay. OT-I alone and GFP-expressing B-cells (GFP-B) were used as control. Results represent the average amount of cytokines from 2-independent experiments with SD (*bars*). **D.** Expression of CD40L on B-cells facilitates the generation of polyfunctional CD8 T-cells. In a parallel experiment with (C), the frequency of cytokine-releasing CD8 T-cells was examined using multi-color intracellular staining against IL-2, IFN-γ, and TNF-α. Results represent the mean percentage of IL-2/IFN-γ- and TNF-α/IFN-γ-double-positive CD8 T-cells from 2-independent experiments with SD (*bars*). Diagrams of the distribution of CD8 T-cells expressing the simultaneously measured cytokines in a representative experiment are presented in right panel of the graphs. P values were calculated using 1-way ANOVA test (*P < 0.05; **P < 0.01; ***P < 0.001).

To enhance the expression of additional costimulatory ligands, B-cells were transduced with CD40L-, CD70-, OX40L-, and 4-1BBL-coding lentivirus alone (CD40L-B, CD70-B, OX40L-B, and 4-1BBL-B-cells, respectively) or co-transduced with CD40L-coding virus together with CD70-, OX40L-, and 4-1BBL-coding virus (CD70/CD40L-B, OX40L/CD40L-B, and 4-1BBL/CD40L-B-cells, respectively), and the surface expression of the recombinant proteins on transduced B-cells was verified (Figure 2A). The levels of the transduced gene expression in B-cells were subsequently increased (more than 50%) in comparison to mock-transduced B-cells. Interestingly, the level of the transduced gene expression was considerably augmented in B-cells that were co-transduced with combinations of viruses encoding individual costimulatory ligands, presumably due to synergistic costimulation of B-cells by dual-transduced costimulatory ligands. Next, we evaluated the antigen-presenting function of genetically modified *ex vivo*-activated B-cells; CFSE-labeled OT-I cells were cultured with variously conditioned B-cells, including dual-transduced costimulatory ligand-expressing B-cells (CD70/CD40L-B-cells) after pulsing with Ova_257_ peptide. Green fluorescent protein-transduced B-cells (GFP-B) were used as control. As shown in Figure 2B, OT-I cells cultured with single costimulatory ligand-expressing B-cells (CD40L-B and CD70-B) had higher cell proliferation indices than GFP-B-cells (proliferation index = 15.8 and 15.2 versus 10.7). Specifically, CD70/CD40L-B-cells enhanced stimulation of OT-I cells (proliferation index = 22.8), suggesting that the expression of CD40L on B-cells benefited CD8 T-cell proliferation *in vitro*. Lastly, cytokine profiles were assessed from supernatants after co-culturing OT-I cells with genetically modified B-cells for 72 h. The levels of IFN-γ, TNF-α, and IL-2 were significantly increased in all co-culture conditions including in GFP-B-cells, where the expression of costimulatory ligand on B-cells benefited cytokine production. IL-4 secretion was not detected under the same conditions (Figure 2C). Accordingly, we assessed the presence of polyfunctional antigen-specific CD8 T-cells using single-cell multi-color staining. As shown in Figure 2D, the expression of CD40L on B-cells facilitated the generation of IL-2/IFN-γ- and TNF-α/IFN-γ-double-positive CD8 T-cells, showing the relative levels of IL-2/IFN-γ/TNF-α-triple-positive polyfunctional CD8 T-cells. However, expression of additional costimulatory ligands on B-cells did not significantly alter cytokine production of B-cells themselves (Supplementary Figure S1), implying that the polarized cytokine production of T-cell responses may be based on enhanced costimulatory signals on antigen-specific T-cells. Overall, these results suggest that the expression of CD40L and CD70 on *ex vivo*-activated B-cells increased antigen-specific CD8 T-cell proliferation *in vitro* through increased type-1 T helper cytokine production.

### Co-expression of CD40L on activated B-cells along with additional costimulatory molecules elicits enhanced CD8 T-cell responses

**Figure 3:**
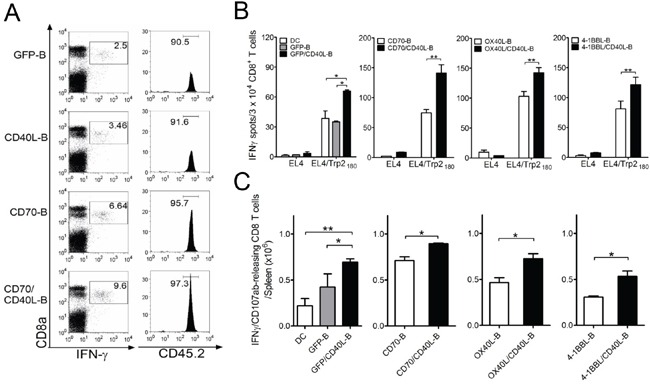
Co-expression of CD40L on *ex vivo*-activated B-cells along with additional costimulatory ligands elicits enhanced CD8 T-cell responses *in vivo* **A.** Congenic (CD45.1) mice received 1 × 10^5^ OT-I (CD45.2) cells 1 day before immunization. Mice were immunized intravenously with variously conditioned B-cells loaded with Ova_257_ peptide. On day 5 post-vaccination, antigen-specific CD8-T-cells in splenocytes revealed by intracellular staining for IFN-γ in representative mice was analyzed by gating for CD45.2-positive cells. Mice vaccinated with GFP-expressing B-cell (GFP-B) were included as control. Numbers in each rectangular gate in the left column represent the percentage of Ova257-specific IFN-γ producing cells of all CD8 T-cells. Numbers in the right column indicate the percentage of CD45.2-positive OT-I cells among all Ova257-specific IFN-γ producing cells. **B.** Mice (3 per group) were immunized intravenously on days 0, and 7 with Trp2180 -loaded B-cells genetically modified to express additional costimulatory ligands as indicated, for comparison of individual or dual expression of costimulatory molecules. Eight days after the last immunization, the presence of antigen-specific CD8 T-cells in spleen was evaluated for their capacity to recognize target cells using IFN-γ EliSpot assay against peptide-pulsed EL4 (EL4/Trp2_180_) and un-pulsed EL4 cells (negative control). Antigen-loaded DCs (DC) and GFP-expressing B-cells (GFP-B) were used as control. Results represent the average number of spots from triplicate wells with SD (*bars*) of the means. **C.** Total numbers of intracellular IFN-γ and cell surface CD107a/b double-positive CD8 T-cells was calculated from the experiment in (B). Splenocytes from each individual mouse were stimulated for cell surface mobilization of CD107a/b and intracellular IFN-γ staining. *Columns*, mean for each group; *bars*, SD. P values were calculated using 1-way ANOVA test (*P < 0.05; **P < 0.01). These experiments were repeated twice with similar results.

To assess whether *ex vivo*-activated B-cells modified to express additional costimulatory ligands can induce antigen-specific CD8 T-cell responses *in vivo*, we examined antigen-specific CD8 T-cell expansion using congenic TCR-transgenic mice. OT-I/CD45.2 cells were infused into congenic CD45.1 mice, followed by immunization with variously conditioned and Ova_257_-pulsed B-cells. As shown in Figure 3A, all B-cell vaccinations led to antigen-specific CD8 T-cell proliferation *in vivo*; CD70/CD40L-B-cell vaccination yielded the highest numbers of antigen-specific CD8 T-cells. Subsequently, mice received three-identical but variously conditioned B-cell vaccinations, and the functional activity of freshly isolated CD8 T-cells (without further *ex vivo* restimulation) was evaluated by IFN-γ EliSpot assays. As shown in Figure 3B and 3C, antigen-specific CD8 T-cell recognition was evident in the peptide-pulsed target (EL4/Trp2_180_), and GFP-B-cell vaccination induced antigen-specific CD8 T-cell responses as efficiently as DC vaccination. The single-gene-modified B-cell (CD40L-B, CD70-B, OX40L-B, and 4-1BBL-B) vaccinations yielded a significantly higher number of IFN-γ spots against target (Figure 3B) and Trp2_180_-specific CD8 T effector cells with lytic functionality (CD107a/b mobilization: Figure 3C) than GFP-B-cell vaccination did. Notably, the mice that received B-cells co-expressing CD40L together with other costimulatory ligands (CD70/CD40L-B, OX40L/CD40L-B, and 4-1BBL/CD40L-B) had significantly higher levels of Trp2_180_-specific CD8 T-cell responses (with lytic functionality) than those receiving other conditioned B-cell vaccinations. Overall, these results indicate that B-cells genetically modified to express additional costimulatory ligands CD70, OX40L, and 4-1BBL exhibit augmented APC function, and additional expression of CD40L enhances their ability to stimulate antigen-specific T-cells *in vivo*.

### Expression of CD40L prolongs the survival of B-cells

**Figure 4:**
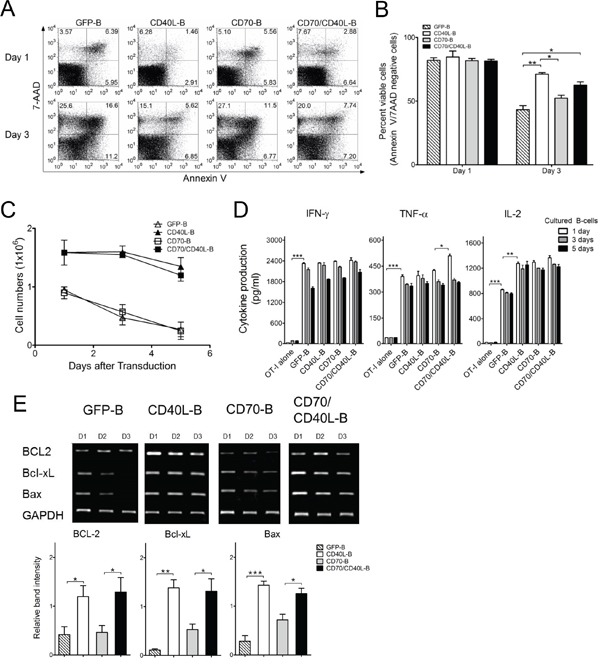
Expression of CD40L prolong the survival of B-cells **A.** Apoptosis in genetically modified B-cells as indicated was examined by staining with annexin-V and 7-AAD at 1 and 3 days post-culturing in the presence of anti-CD40 Antibodies and IL-4. Results represent representative experiments of annexin-V/7-AAD levels assessed by flow cytometry. **B.** The mean percentage of annexin-V and 7-AAD-negative cells (viable cells) was calculated from 2-independent experiments. *bars*, SD. **C.** The viable cell counts were determined on day 1, 3 and 5 days post-culturing from the experiment in (B). *Points*, mean values of fold expansion over time; *bars*, SD. **D.** Genetically modified B-cells maintained intact APC function. *Ex vivo*-generated B-cells were maintained for 1, 3, and 5 days in the presence of 10 μg/mL anti-CD40 antibodies and 10 ng/mL IL-4. Cytokine profiles were evaluated in the same manner as described in Figure 2C. Results represent the average amount of cytokines from 2-independent experiments with SD (*bars*). **E.** Reverse transcription-PCR analysis of apoptosis-related molecules BCL2, Bcl-xL and Bax. Three days post-transduction, the mRNA were obtained from variously conditioned B-cells. Intensity of the bands was measured by Image Lab software and the mRNA expression levels were normalized to that of GAPDH. *Lower panel*, Results represent the mean intensity of the bands from 3-independent experiments with SD (*bars*). P values were calculated using 1-way ANOVA test (*P < 0.05; **P < 0.01; ***P < 0.001).

Insufficient endurance of infused APC cells *in vivo* has been suggested as an explanation for the inefficient induction of antigen-specific CD8 T-cells. The CD40L:CD40 interaction in B-cells is known to be crucial for the generation of long-lived plasma cells and memory B-cells, as well as for their survival [21, 22]. In view of this, we analyzed the survival rate of variously conditioned costimulatory ligand-transduced B-cells. The CD40L- and CD70/CD40L-expressing B-cells prevented spontaneous cell death more efficiently than GFP- and CD70-expressing B-cells did (Figure 4A and 4B), resulting in higher numbers of live CD40L-expressing B-cells. In contrast, the number of CD40L-lacking B-cells (GFP-B and CD70-B) declined to about 2-fold less than that of CD40L-presenting B-cells (Figure 4C). Nevertheless, it should be mentioned that the APC function of genetically modified B-cells remained intact even in 5-days-cultured B-cells post-transduction (Figure 4D). Similar to the results of the cell survival studies, B-cells transduced with CD40L (CD40L-B and CD70/CD40L-B) allowed the long-term persistence of anti-apoptotic molecules BCL2, Bcl-xL and Bax better than CD40L-lacking B-cells (Figure 4E). These results indicate that expression of CD40L in B-cells enhanced *ex vivo* viability and inhibited apoptosis *in vivo* by increasing expression of anti-apoptotic proteins.

### Enhancement of therapeutic efficacy with B-cells co-expressing CD40L and other costimulatory ligands

**Figure 5:**
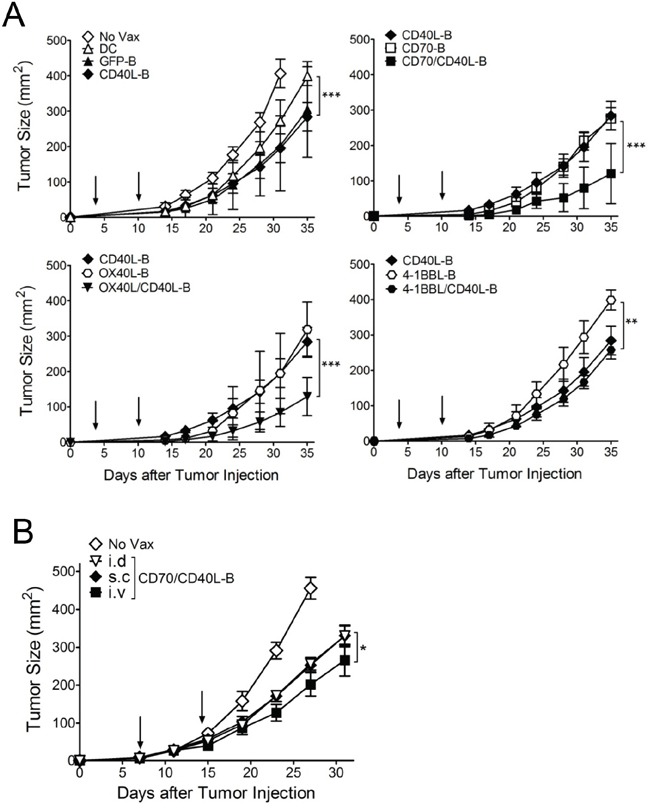
Therapeutic antitumor effects of antigen-loaded B-cells expressing costimulatory ligands against established B16 melanoma **A.** Effects of costimulatory ligands on the therapeutic efficacy of *ex vivo*-activated B-cell vaccines transduced with costimulatory ligands either individually or in pairs as indicated. B6 mice (5 per group) were inoculated subcutaneously on day 0 with 1 × 10^5^ B16 cells, and vaccinated intravenously on day 3, and 10 (*vertical arrow*) with Trp2180 -loaded B-cells. **B.** Effects of the mode of *ex vivo*-generated B-cells administration. B6 mice (4 per group) were inoculated subcutaneously on day 0 with 3 × 10^5^ B16 cells and received Trp2_180_ -loaded B-cells through different routes on day 7, and 14 (*vertical arrow*). Trp2_180_-loaded CD70/CD40L-B-cells were administered intradermally (i.d), subcutaneously (s.c), or intravenously (i.v). Non-vaccinated mice (No Vax) and antigen-loaded DCs (DC) were included as controls. Tumor sizes were determined in individual mice by measurements of two opposing diameters and are presented as tumor areas in mm^2^. *Points*, mean for each group of mice; *bars*, SD. *P* values were calculated using 2-way ANOVA test (*P < 0.05; **P < 0.01; ***P < 0.001). These experiments were repeated twice with similar results.

**Figure 6:**
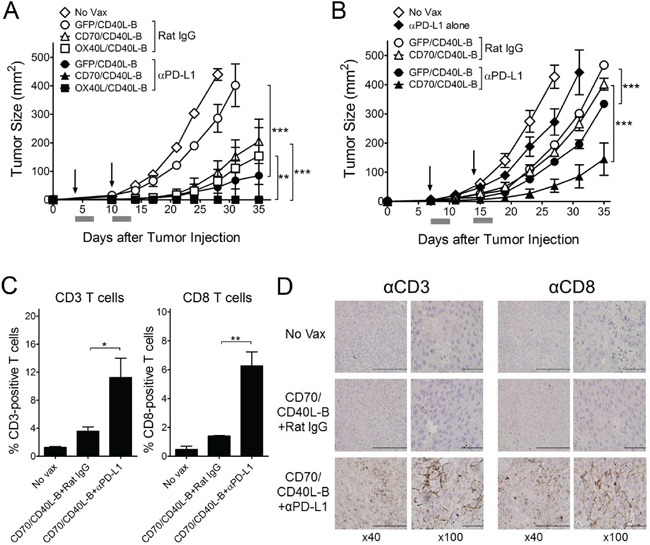
PD-1 blockade enhance the therapeutic efficacy of *ex vivo*-generated B-cell vaccines **A.** Effects of PD-1 blockade on the therapeutic efficacy of B-cell vaccines transduced with costimulatory ligands in pairs as indicated. B6 mice (5 per group) were inoculated subcutaneously on day 0 with 1 × 10^5^ B16 cells, and vaccinated intravenously on day 3, and 10 (*vertical arrow*) with Trp2180 -loaded B-cells. No Vax and GFP/CD40L-B-cell-vaccinated mice were included as control. **B.** Therapeutic effects of genetically modified B-cells against advanced B16 tumors. B6 mice (5 per group) were inoculated subcutaneously on day 0 with 3 × 10^5^ B16 cells, followed by vaccination with B-cell vaccines on days 7, and 14 (*vertical arrow*). A group of mice administered anti-PD-L1 only (αPD-L1 alone) was included to verify the therapeutic efficacy of *ex vivo*-generated B-cell vaccines. Anti-PD-L1 and normal rat IgG were administered as described *in Materials and Methods*. Tumor sizes were determined in individual mice by measurements of two opposing diameters and are presented as tumor areas in mm^2^. *Gray bars*, time period of PD-1 blockade. *Points*, mean for each group of mice; *bars*, SD. P values were calculated using 2-way ANOVA test (**P < 0.01; ***P < 0.001). These experiments were repeated twice with similar results. **C** and **D.** PD-1 blockade elicits improved levels of T-cells in tumor sites. B6 mice (3 per group) were inoculated subcutaneously on day 0 with 3 × 10^5^ B16 cells, and immunized intravenously on days 7 and 14 with Trp2_180_-loaded CD70/CD40L-B-cells. Tumors were excised on day 8 after the last immunization, and the infiltration of CD3- or CD8-positive T-cells was evaluated using flow cytometry (C) and immunohistochemical analysis (D). *C*, Results represent the mean percentage of CD3- or CD8-positive T-cells from individual mice with SD (*bars*). *P* values were calculated using 1-way ANOVA test (*P < 0.05; **P < 0.01). *D*, Representative images show CD3- or CD8-positive T-cell infiltration in tumor tissues with x40 and x100 magnification (*brown*). *Scale bar*, 200 μm in x40 and 50 μm in x400.

Next, we assessed whether CD8 T-cells generated with genetically modified B-cell vaccinations are capable of triggering *in vivo* therapeutic antitumor effects against 3-day established tumors. As shown in Figure 5A, the single-gene-modified B-cell (CD40L-B, CD70-B, OX40L-B, and 4-1BBL-B) vaccinations had moderate therapeutic effects that were superior to DC vaccination or GFP-B-cell vaccination. In contrast, B-cells co-expressing CD40L and the other costimulatory ligands except 4-1BBL (CD70/CD40L-B and OX40L/CD40L-B) had substantially higher antitumor effects (2 out of 5 mice completely rejected the tumors in the CD70/CD40L-B and OX40L/CD40L-B groups). Mice that received CD70/CD40L-B and OX40L/CD40L-B-cells survived more than 90 days (data not shown). To assess route of *ex vivo*-generated B-cell delivery, antigen-loaded CD70/CD40L-B-cells were infused through different routes into B16-tumor bearing mice. The results shown in Figure 5B indicate that both subcutaneous and intradermal routes of B-cell vaccination were highly effective and comparable for the therapeutic antitumor immunity while the intravenous route was even more efficient. Subsequently, we evaluated the roles of PD-1 blockade (with anti-PD-L1 antibodies), which could cooperate with CD8 T-cells in fighting established tumors. As shown in Figure 6A, PD-1 blockade potentiated the therapeutic efficacy of genetically modified B-cells, where a slight increase in antigen-specific CD8 T-cells was observed from peripheral blood samples on day 32 post-tumor inoculation (data not shown). Obviously, the administration of isotype control rat IgG had no effects on tumor growth (Supplementary Figure S2). Furthermore, tumor size may impact the therapeutic efficacy of cancer immunotherapy; therefore, we evaluated the therapeutic benefits of genetically modified B-cell vaccination against 7-day established tumors (3-5 mm in diameter). Under these circumstances, vaccination with CD40L-expressing B-cells reduced the median tumor growth rate by approximately 7 days (Figure 6B). Although the administration of anti-PD-L1 antibodies alone had a moderate therapeutic effect, the addition of PD-1 blockade to the B-cell vaccines significantly reduced the median tumor growth rate compared to the non-combined groups, but no tumor rejections were observed. To evaluate the effector T-cells involved in the antitumor effects with PD-1 blockade, flow cytometric and immunohistochemical analyses were performed to detect invading the T-cells into the tumor tissues. The results presented in Figure 6C and 6D show addition of anti-PD-L1 antibodies resulted in augmenting the frequency of CD3 T-cell (comprising CD8 T-cells) infiltration into the tumor site, suggesting that PD-1 blockade affects the persistence and function of tumor-infiltrating lymphocytes in the tumor microenvironment. More interestingly, the CD8 T-cells from tumor-bearing mice that received Trp2_180_-pulsed CD70/CD40L-B-cells were effective against other melanoma-associated antigens Trp1 and gp100 (Supplementary Figure S3), indicating that genetically modified B-cell immunization in tumor-bearing mice may effectively generate epitope spreading. Overall, these results indicate that vaccination with B-cells genetically modified to express additional costimulatory ligands was effective in circumventing any potential tolerance to self-antigens expressed by normal tissues, resulting in significant antitumor therapeutic effects against established tumors.

## DISCUSSION

It is evident that naïve T-cell priming and activation depend in great part on costimulatory signals derived from CD80, CD86, CD70, OX40L, and 4-1BBL expressed on APCs and sufficient interaction of TCRs with the pMHC complex [3, 23]. Manipulation of costimulatory ligands on cells has been shown to confer protective immunity against subsequent tumor challenge by inducing effective T-cell responses in mice, implying that sustained expression of one or multiple costimulatory molecules can enhance APC function and the resulting T-cell responses [24–26].

The purpose of the present study was to assess the effectiveness of genetically modified B-cells co-expressing CD40L in combination with CD70, OX40L, and 4-1BBL to provide costimulatory signals to CD8 T-cells, as an alternative source of autologous APCs for cancer immunotherapy. Administration of *ex vivo*-activated B-cells co-expressing CD40L along with other costimulatory ligands CD70 and OX40L gave substantial antitumor effects in a B16 melanoma model. In the clinical realm, existing DC-based cancer immunotherapy efforts have yielded encouraging but inconsistent clinical outcomes [10]. To overcome the significant drawbacks of DC-based vaccine design, we and others have investigated multimodality strategies to engineer DCs to improve their maturation, migration, and longevity in order to enhance APC function and efficacy [27–29]. Several studies have shown that expression of costimulatory ligands CD40L, CD70, OX40L, and 4-1BBL on DCs overcomes T-cell tolerance and establishes superior protective immunity to tumor challenge or viral infection. Specifically, the engagement of CD40 by CD40L on DCs has been shown to promote cytokine production and upregulation of costimulatory molecules, involving optimal T-cell activation and differentiation [6, 30, 31].

*Ex vivo*-activated B-cells, which are capable of presenting exogenous antigens to activate T-cell responses, have been potentiated as therapeutic APCs because they can be obtained in large numbers from a small volume of peripheral blood, and are easily expanded *in vitro* to yield a pure and homogeneous population [32]. In this respect, earlier studies have shown that antigen-loaded B-cells, which were activated *in vitro* with inflammatory cytokines, CD40L, and TLR ligands, are capable of enhancing T-cell stimulatory capacity and inducing protective immunity *in vivo* [14, 18]. However, additional concerns have been raised over the use of B-cell vaccines for cancer immunotherapy because a variety of studies have demonstrated that activated B-cells are less effective stimulators of T-cells than DCs, presumably due to insufficient costimulatory ligands on B-cells [14, 33]. A recent study reported that multiple RNA-transfected B-cells expressing exogenous OX40L, 4-1BBL, and IL-12 induced antigen-specific T-cell responses as efficiently as mature DCs *in vitro*, but produced less antitumor immunity *in vivo* [19]. Nonetheless, our results demonstrate that CD40L-B-cell vaccination could induce superior CD8 T-cell responses and antitumor immunity against B16 melanoma compared to DC vaccination (Figures 3B and 5A), indicating that additional expression of CD40L molecules on B-cells achieves enhanced APC function to stimulate antigen-specific T-cells *in vivo*.

Numerous reports have shown the successful *ex vivo* expansion of B-cells in the presence of B-cell growth factors and CD40 signals such as use of anti-CD40 antibodies, recombinant soluble CD40L, or CD40L-expressing cell lines [34, 35]. The interaction of CD40L with CD40 on B-cells plays an essential role in the generation of long-lived plasma cells and memory B-cells, as well as in their long-term survival *in vivo* [21, 22]. Sustained B-cell receptor signaling is essential for their survival during consecutive cell divisions where engagement of CD40, mimicking contact-dependent T-cell help, can selectively rescue defects in B-cell survival and proliferation after poor B-cell receptor stimulation [36, 37]. In a report by Wilker and colleagues [38], engagement of CD40 restored the survival of myocyte-enhancer factor 2c-deficient B-cells, which have a substantial defect in expression of Bcl-xL, resulting in poor proliferation and survival in response to B-cell receptor stimulation. We also observed that the expression of CD40L on B-cells augmented cell viability by upregulating the anti-apoptotic molecules BCL2, Bcl-xL and Bax, resulting in improved APC function *in vitro* and *in vivo* (Figure 4E). There is some evidence that cell longevity *in vivo* is an important factor in the long-term ability of APCs to stimulate antigen-specific T- cells. In view of this, genetic modifications that upregulate anti-apoptotic signaling or reduce pro-apoptotic signaling have been investigated to enhance the survival of DCs for cancer immunotherapy in preclinical models [39, 40]. We have also reported that the expression of costimulatory ligands CD80 and 4-1BBL on murine CD4 T-cells prolonged their longevity *in vitro* and *in vivo*, indicating their potential for use as alternative APCs for cancer immunotherapy [12]. In a report by Stephan and colleagues [41], human T-cells co-expressing CD80 and 4-1BBL exhibited superior *in vitro* proliferation and survival by interacting with their respective receptors in the immunological synapse of isolated single cells (auto-costimulation) as well as in bystander cells (trans-costimulation). These results imply that the expression of CD40L on B-cells augmented not only conventional costimulation of antigen-priming T-cells but also increased cell viability through both auto- and trans-costimulation, significantly increasing therapeutic benefits in preclinical settings.

More importantly, our results show that the single or dual expression of costimulatory ligands CD40L, CD70, and OX40L on B-cells potentiated their APC functions, thereby enhancing antitumor immunity. However, it remains unknown why the expression of 4-1BBL, even with CD40L, was not as effective as the other costimulatory ligands (Figure 5A). Many routes of vaccine delivery have been tested, each with positive and negative aspects to consider [42]. In previous studies, we observed that superior antigen-specific T-cell responses were obtained when antigen-loaded B-cell-based vaccines were administered via an intravenous route compared with the subcutaneous route [15]. Likewise, our results indicated that both the subcutaneous and intradermal injections of genetically modified B-cell vaccination were highly effective and comparable in their therapeutic antitumor immunity; intravenous injections were even efficient (Figure 5B). Nonetheless, route of *ex vivo*-generated B-cell delivery still remains to be defined for human clinical trials because it would be extremely difficult to achieve intranodal or intralymphatic administration of vaccines in mice. Our results also show that addition of anti-PD-L1 antibodies further enhanced the antitumor effects of the genetically-modified B-cell vaccine even in advanced B16 tumors (Figure 6A and 6B). It has recently become clear that multiple immune inhibitory mechanisms are present in tumor sites and PD-1 blockade prevents exhaustion of tumor-infiltrating T-cells, leading to augmented effector function and persistence of antigen-specific T-cells at the tumor site [43, 44]. We also observed that PD-1 blockade led to increase the frequency of tumor-infiltrating T-cells in the tumor sites, resulting in enhancing antitumor efficacy with the increase in overall T-cell numbers (Figure 6C and 6D). PD-L1 is widely expressed on immune cells such as macrophages, DCs, T-, and B-cells, and upregulated in response to inflammatory stimuli [45]. A recent publication by Pilon-Thomas and colleagues showed that PD-L1 was expressed on murine bone marrow-derived DCs, and blockade of PD-L1 interaction on DCs enhanced cytolytic activities of antigen-specific T-cells *in vitro* and increased the number of antigen-specific CD8 T-cells *in vivo* [46]. Likewise, we observed PD-L1 expression on *ex vivo*-activated B-cells (data not shown), presumably indicating that the systemic administration of anti-PD-L1 antibodies prevents exhaustion of not only the tumor-infiltrating antigen-specific T-cells but also infused antigen-loaded B-cells to preserve prolonged APC function.

Collectively, our data show that B-cells genetically manipulated to express CD40L along with CD70 or OX40L display augmented APC function and cell viability, resulting in remarkable therapeutic antitumor benefits, suggesting that the genetically modified B-cells could serve as an alternative source of autologous APCs to overcome the limitation of DC-based therapeutic vaccines. Furthermore, the results in mouse tumor models demonstrate the applicability of the genetically modified B-cell vaccination strategy to the treatment of a broad variety of malignancies and viral diseases.

## MATERIALS AND METHODS

### Mice

C57BL/6 (B6) mice were purchased from Orient Bio (Seongnam, Korea). B6.SJL congenic (CD45.1) and OT-I TCR-transgenic mice were obtained from Jackson Laboratories (Bar Harbor, ME), and bred in our animal facilities under pathogen-free conditions. All animal research was conducted in accordance with our institutional animal care and use committee guidelines.

### Cell lines, peptides, and reagents

Murine melanoma B16F10, 293T, and EL4 cells were obtained from the American Type Culture Collection (Manassas, VA). All cell lines were cultured as recommended by the provider. Synthetic peptides representing the CD8 T-cell epitopes Ova_257_ (SIINFEKL) and Trp2_180_ (SVYDEFVWL) were purchased at >80% purity from A&A Labs (San Diego, CA). Monoclonal anti-mouse CD40 (FGK45.5) and anti-PD-L1 (10F.9G2) were purchased from BioXCell (West Lebanon, NH). Fluorescence-conjugated antibodies for flow cytometry were from eBioscience (San Diego, CA).

### Production of recombinant lentiviruses encoding costimulatory ligands

The cDNAs for mouse CD40L, CD70, OX40L, 4-1BBL were amplified by reverse transcription (RT)-PCR from total RNA extracted from matured DCs using the specific primers listed in Supplementary Table S1. The PCR products were cloned into the lentiviral vector pCDH-EF1 (System Bioscience, Palo Alto, CA) with *Bsp*EI and *Sal*I sites, which has a constitutive elongation factor 1α promoter for transcription of cloned cDNA insert, and sequenced to determine the possible Taq polymerase errors. Schematic diagram of the constructs are shown in Supplementary Figure S4. For production of recombinant lentiviruses encoding the above costimulatory ligands, 7 × 10^6^ 293T cells were seeded in a 100 mm culture plate coated with 5 μg/ml of poly-Lysine (Sigma, St. Louis, MO). Twenty hours later, 12 μg a cloned pCDH plasmid and lentivirus packaging plasmids (8 μg psPAX2 and 4 μg pMD2G) were simultaneously transfected into 293T cells using lipofectamine (Invitrogen, Carlsbad, CA), according to the manufacturer's **instructions**. Two days later, the recombinant lentiviruses were harvested and titrated into 293T cells, which were then used for the transduction experiments.

### Transduction recombinant lentiviruses encoding costimulatory ligands into *ex vivo*-activated B-cells

B-cells were obtained from spleens of B6 mice using MACS anti-CD43 microbeads (Miltenyi Biotec, Auburn, CA), resulting in >90% purity. For *in vitro* pre-activation, purified B-cells were cultured at 2 × 10^6^ cells per well in 24-well plates in the presence of 10 μg/mL anti-CD40 antibodies and 10 ng/mL IL-4 (PeproTech, Rocky Hill, NJ). Twenty hours later, activated B-cells were harvested and seeded at 2 × 10^6^ cells per well in 24-well plates, followed by adding recombinant lentiviruses encoding costimulatory ligands either individually (MOI = 0.5) or in pairs as indicated with twice the amount of virus for co-transduction (eventually, MOI = 1). Cells were centrifuged at 1800 rpm for 30 min at 25°C in the presence of 5 μg/mL polybrene (Sigma) and additionally incubated for 1 h in a 37°C /5% CO_2_ incubator, followed by replacement with the above-described culture medium containing a 10 μg/mL peptide (Ova_257_ or Trp2_180_). Twenty hours later, genetically modified and antigen-loaded B-cells were used for vaccination in most instances.

### Assessment of *in vitro* apoptosis and cell viability with RT-PCR

For *in vitro* apoptosis, the genetically modified B-cells were cultured in the presence of 10 μg/mL anti-CD40 antibodies and 10 ng/mL IL-4. On day 1, 3, and 5, the cells were analyzed by annexin-V and 7-amino-actinomycin D (7-AAD) staining. Total cell counts were performed with trypan blue exclusion. For RT-PCR analysis of apoptosis-related molecules, total RNA was extracted from variously conditioned B-cells using an RNeasy Kit (Qiagen, Valencia, CA) and cDNA was synthesized using a First Strand cDNA Synthesis Kit (Roche, Basel, Switzerland). Subsequently, quantitative RT-PCR analysis for apoptosis-related mRNAs was performed using the primers listed in Supplementary Table S2. The thermocycling program was as follows: 95°C for 4 min, 35 cycles of 95°C for 30 s, 62.8°C for 30 s, 72°C for 60 s, and 10 min at 72°C for a final amplicon extension. Intensity of the bands was measured by Image Lab software (Bio-Rad, Hercules, CA) and the mRNA expression relative to that of GAPDH was calculated for each sample.

### *In vitro/in vivo* proliferation assays and measurement of cytokine production

OT-I cells purified using MACS anti-CD8-micobeads (Miltenyi Biotec) were labeled with carboxyfluorescein succinimidyl ester (CFSE; Molecular Probes, Eugene, OR) at a final concentration of 5 μM for 15 min. For *in vitro* proliferation, 2 × 10^5^ OT-I cells were incubated with 1 × 10^5^ genetically modified and Ova_257_-loaded B-cells in 24-well plates. Four days later, cells were stained with anti-CD8 antibodies and analyzed. Fluorescence was measured using a FACS Calibur flow cytometer (BD Biosciences, San Jose, CA) and analyzed using FlowJo software. Cell proliferation was calculated using the Proliferation Wizard Model of Modfit LT software (Verity Software House, Topsham, ME). At the same time, the supernatants were harvested and stored at −70°C for further examination of cytokine production. The concentration of cytokines in the supernatants was determined with an ELISA kit (eBioscience), according to the manufacturer's instructions. For *in vivo* proliferation, 1 × 10^5^ OT-I/CD45.2 cells were administered intravenously to congenic (CD45.1) mice. On day 1 after OT-I cell infusion, mice received 2 × 10^6^ genetically modified and Ova_257_-loaded B-cells as indicated. On day 5 post-vaccination, splenocytes were incubated with 1 μg/mL Ova_257_ peptide for 18 h and subsequently subjected to intracellular IFN-γ staining using anti-CD8 and anti-CD45.2 antibodies.

### Immunizations and evaluation of immune responses

Mice were immunized intravenously with 2 × 10^6^ Trp2_180_-loaded B-cells expressing additional costimulatory ligands as indicated. Mice received an identical booster immunization after a 7 day interval. Some mice were immunized intravenously with 2 × 10^6^ DCs pulsed with 10 μg/mL Trp2_180_ for 18 h. DCs were generated from bone marrow monocytes cultured for 6 days with 10 ng/mL GM-CSF (PeproTech) and 5 ng/mL IL-4. For measuring antigen-specific CD8 T-cell responses, splenocytes were incubated with 1 μg/mL peptide and 1 μl/mL GolgiPlug (BD Bioscience) at 37°C. After 6 h, cells were stained for intracellular IFN-γ following the directions provided by the vendor (BD Bioscience) using fluorescence-conjugated antibodies against MHC class II, CD8a, and IFN-γ. For CD107a/b mobilization shift assay, 2.5 μg/mL of fluorescence-conjugated anti-CD107a and CD107b antibodies were added at the beginning of the stimulation period. For the *in vitro* T-cell recognition, IFN-γ enzyme-linked immunosorbent spot (EliSpot) assays were performed using freshly isolated CD8 T-cells (Miltenyi Biotec) from spleens, as described previously [5]. Peptide-pulsed or un-pulsed EL4 cells were used as targets cells.

### Evaluation of therapeutic antitumor effects

Mice were inoculated subcutaneously with 1 × 10^5^ B16 melanoma cells in the rear flank, and 3 days later the first immunization was administered intravenously with 2 × 10^6^ Trp2_180_-loaded B-cells expressing additional costimulatory ligands as indicated. In some instances, mice were inoculated with 3 × 10^5^ B16 cells (for advanced tumor model), and vaccinated 7 days later. For PD-1 blockade, anti-PD-L1 and control rat IgG were administered intraperitoneally (200 μg/dose) on days 0, 2, and 4 after each immunization (prime and boost). Tumor growth was monitored every 3–4 d in individual tagged mice by measuring two opposing diameters with a set of calipers. Mice were euthanized when the tumor area reached >400 mm^2^. Results are presented as mean tumor size (area in mm^2^) ± SD for every treatment group at various time points until the termination of the experiment.

### Statistical analyses

Statistical significance to assess numbers of antigen-specific CD8 T-cells and tumor sizes were determined using 1-way and 2-way ANOVA test, respectively. The results are representative of data obtained from at least 2 independent experiments. All analyses were performed and graph made using Prism 5.01 software (GraphPad, San Diego, CA).

## SUPPLEMENTARY FIGURES AND TABLES



## References

[1] Cerottini JC, Brunner KT (1974). Cell-mediated cytotoxicity, allograft rejection, and tumor immunity. Advances in immunology.

[2] Dougan M, Dranoff G (2009). Immune therapy for cancer. Annual review of immunology.

[3] Croft M (2003). Costimulatory members of the TNFR family: keys to effective T-cell immunity?. Nature reviews Immunology.

[4] Garrigan K, Moroni-Rawson P, McMurray C, Hermans I, Abernethy N, Watson J, Ronchese F (1996). Functional comparison of spleen dendritic cells and dendritic cells cultured *in vitro* from bone marrow precursors. Blood.

[5] Cho HI, Jung SH, Sohn HJ, Celis E, Kim TG (2015). An optimized peptide vaccine strategy capable of inducing multivalent CD8 T-cell responses with potent antitumor effects. Oncoimmunology.

[6] Liu Y, Zhang X, Zhang W, Chen Z, Chan T, Ali K, Jia Z, Xiang J (2002). Adenovirus-mediated CD40 ligand gene-engineered dendritic cells elicit enhanced CD8(+) cytotoxic T-cell activation and antitumor immunity. Cancer gene therapy.

[7] Mende I, Engleman EG (2005). Breaking tolerance to tumors with dendritic cell-based immunotherapy. Annals of the New York Academy of Sciences.

[8] Ardavin C, Amigorena S, Reis e Sousa C (2004). Dendritic cells: immunobiology and cancer immunotherapy. Immunity.

[9] Radford KJ, Tullett KM, Lahoud MH (2014). Dendritic cells and cancer immunotherapy. Current opinion in immunology.

[10] Turnis ME, Rooney CM (2010). Enhancement of dendritic cells as vaccines for cancer. Immunotherapy.

[11] Himoudi N, Morgenstern DA, Yan M, Vernay B, Saraiva L, Wu Y, Cohen CJ, Gustafsson K, Anderson J (2012). Human gammadelta T lymphocytes are licensed for professional antigen presentation by interaction with opsonized target cells. Journal of immunology.

[12] Park HM, Sohn HJ, Kim YJ, Cho HI, Kim TG (2014). CD4 T-cells transduced with CD80 and 4-1BBL mRNA induce long-term CD8 T-cell responses resulting in potent antitumor effects. Vaccine.

[13] Penafuerte C, Ng S, Bautista-Lopez N, Birman E, Forner K, Galipeau J (2012). B effector cells activated by a chimeric protein consisting of IL-2 and the ectodomain of TGF-beta receptor II induce potent antitumor immunity. Cancer research.

[14] Schultze JL, Michalak S, Seamon MJ, Dranoff G, Jung K, Daley J, Delgado JC, Gribben JG, Nadler LM (1997). CD40-activated human B-cells: an alternative source of highly efficient antigen presenting cells to generate autologous antigen-specific T-cells for adoptive immunotherapy. The Journal of clinical investigation.

[15] Park MY, Kim HS, Woo SJ, Kim CH, Park JS, Sohn HJ, Kim HJ, Oh ST, Kim TG (2008). Efficient antitumor immunity in a murine colorectal cancer model induced by CEA RNA-electroporated B-cells. European journal of immunology.

[16] Guo S, Xu J, Denning W, Hel Z (2009). Induction of protective cytotoxic T-cell responses by a B-cell-based cellular vaccine requires stable expression of antigen. Gene therapy.

[17] Kim YJ, Ko HJ, Kim YS, Kim DH, Kang S, Kim JM, Chung Y, Kang CY (2008). alpha-Galactosylceramide-loaded, antigen-expressing B-cells prime a wide spectrum of antitumor immunity. International journal of cancer.

[18] Chung Y, Kim BS, Kim YJ, Ko HJ, Ko SY, Kim DH, Kang CY (2006). CD1d-restricted T-cells license B-cells to generate long-lasting cytotoxic antitumor immunity *in vivo*. Cancer research.

[19] Lee J, Dollins CM, Boczkowski D, Sullenger BA, Nair S (2008). Activated B-cells modified by electroporation of multiple mRNAs encoding immune stimulatory molecules are comparable to mature dendritic cells in inducing *in vitro* antigen-specific T-cell responses. Immunology.

[20] Ren H, Zhao S, Li W, Dong H, Zhou M, Cao M, Hu HM, Wang LX (2014). Therapeutic antitumor efficacy of B-cells loaded with tumor-derived autophagasomes vaccine (DRibbles). Journal of immunotherapy.

[21] Bolduc A, Long E, Stapler D, Cascalho M, Tsubata T, Koni PA, Shimoda M (2010). Constitutive CD40L expression on B-cells prematurely terminates germinal center response and leads to augmented plasma cell production in T-cell areas. Journal of immunology.

[22] Clark LB, Foy TM, Noelle RJ (1996). CD40 and its ligand. Advances in immunology.

[23] Croft M (2003). Costimulation of T-cells by OX40, 4-1BB, and CD27. Cytokine & growth factor reviews.

[24] Driessens G, Kline J, Gajewski TF (2009). Costimulatory and coinhibitory receptors in antitumor immunity. Immunological reviews.

[25] Johnson BD, Gershan JA, Natalia N, Zujewski H, Weber JJ, Yan X, Orentas RJ (2005). Neuroblastoma cells transiently transfected to simultaneously express the costimulatory molecules CD54, CD80, CD86, and CD137L generate antitumor immunity in mice. Journal of immunotherapy.

[26] Townsend SE, Allison JP (1993). Tumor rejection after direct costimulation of CD8+ T-cells by B7-transfected melanoma cells. Science.

[27] Datta J, Terhune JH, Lowenfeld L, Cintolo JA, Xu S, Roses RE, Czerniecki BJ (2014). Optimizing dendritic cell-based approaches for cancer immunotherapy. The Yale journal of biology and medicine.

[28] Boudreau JE, Bonehill A, Thielemans K, Wan Y (2011). Engineering dendritic cells to enhance cancer immunotherapy. Molecular therapy.

[29] Cho HI, Kim EK, Park SY, Lee SK, Hong YK, Kim TG (2007). Enhanced induction of antitumor immunity in human and mouse by dendritic cells pulsed with recombinant TAT fused human survivin protein. Cancer letters.

[30] De Keersmaecker B, Heirman C, Corthals J, Empsen C, van Grunsven LA, Allard SD, Pen J, Lacor P, Thielemans K, Aerts JL (2011). The combination of 4-1BBL and CD40L strongly enhances the capacity of dendritic cells to stimulate HIV-specific T-cell responses. Journal of leukocyte biology.

[31] Yurkovetsky ZR, Shurin GV, Barry DA, Schuh AC, Shurin MR, Robbins PD (2006). Comparative analysis of antitumor activity of CD40L, RANKL, and 4-1BBL *in vivo* following intratumoral administration of viral vectors or transduced dendritic cells. The journal of gene medicine.

[32] Schultze JL, Grabbe S, von Bergwelt-Baildon MS (2004). DCs and CD40-activated B-cells: current and future avenues to cellular cancer immunotherapy. Trends in immunology.

[33] Cassell DJ, Schwartz RH (1994). A quantitative analysis of antigen-presenting cell function: activated B-cells stimulate naive CD4 T-cells but are inferior to dendritic cells in providing costimulation. The Journal of experimental medicine.

[34] Wennhold K, Shimabukuro-Vornhagen A, Theurich S, von Bergwelt-Baildon M (2013). CD40-activated B-cells as antigen-presenting cells: the final sprint toward clinical application. Expert review of vaccines.

[35] Zheng J, Liu Y, Qin G, Chan PL, Mao H, Lam KT, Lewis DB, Lau YL, Tu W (2009). Efficient induction and expansion of human alloantigen-specific CD8 regulatory T-cells from naive precursors by CD40-activated B-cells. Journal of immunology.

[36] Donahue AC, Fruman DA (2003). Proliferation and survival of activated B-cells requires sustained antigen receptor engagement and phosphoinositide 3-kinase activation. Journal of immunology.

[37] Zarnegar B, He JQ, Oganesyan G, Hoffmann A, Baltimore D, Cheng G (2004). Unique CD40-mediated biological program in B-cell activation requires both type 1 and type 2 NF-kappaB activation pathways. Proceedings of the National Academy of Sciences of the United States of America.

[38] Wilker PR, Kohyama M, Sandau MM, Albring JC, Nakagawa O, Schwarz JJ, Murphy KM (2008). Transcription factor Mef2c is required for B-cell proliferation and survival after antigen receptor stimulation. Nature immunology.

[39] Hou WS, Van Parijs L (2004). A Bcl-2-dependent molecular timer regulates the lifespan and immunogenicity of dendritic cells. Nature immunology.

[40] Park D, Lapteva N, Seethammagari M, Slawin KM, Spencer DM (2006). An essential role for Akt1 in dendritic cell function and tumor immunotherapy. Nature biotechnology.

[41] Stephan MT, Ponomarev V, Brentjens RJ, Chang AH, Dobrenkov KV, Heller G, Sadelain M (2007). T-cell-encoded CD80 and 4-1BBL induce auto- and transcostimulation, resulting in potent tumor rejection. Nature medicine.

[42] Butterfield LH (2013). Dendritic cells in cancer immunotherapy clinical trials: are we making progress?. Frontiers in immunology.

[43] Duraiswamy J, Kaluza KM, Freeman GJ, Coukos G (2013). Dual blockade of PD-1 and CTLA-4 combined with tumor vaccine effectively restores T-cell rejection function in tumors. Cancer research.

[44] Mumprecht S, Schurch C, Schwaller J, Solenthaler M, Ochsenbein AF (2009). Programmed death 1 signaling on chronic myeloid leukemia-specific T-cells results in T-cell exhaustion and disease progression. Blood.

[45] Sharpe AH, Wherry EJ, Ahmed R, Freeman GJ (2007). The function of programmed cell death 1 and its ligands in regulating autoimmunity and infection. Nature immunology.

[46] Pilon-Thomas S, Mackay A, Vohra N, Mule JJ (2010). Blockade of programmed death ligand 1 enhances the therapeutic efficacy of combination immunotherapy against melanoma. Journal of immunology.

