# Supplementary material for: Co-expression of CD40L with CD70 or OX40L increases B-cell viability and antitumor efficacy
Source: Oncotarget. 2016 Jun 15;7(29):46173–86. doi: 10.18632/oncotarget.10068 (PMC5216789; doi:10.18632/oncotarget.10068)
Supplement: Supplementary file 1 [file oncotarget-07-46173-s001.pdf]

# Co-expression of CD40L with CD70 or OX40L increases B-cell viability and antitumor efficacy

## SUPPLEMENTARY FIGURES AND TABLES

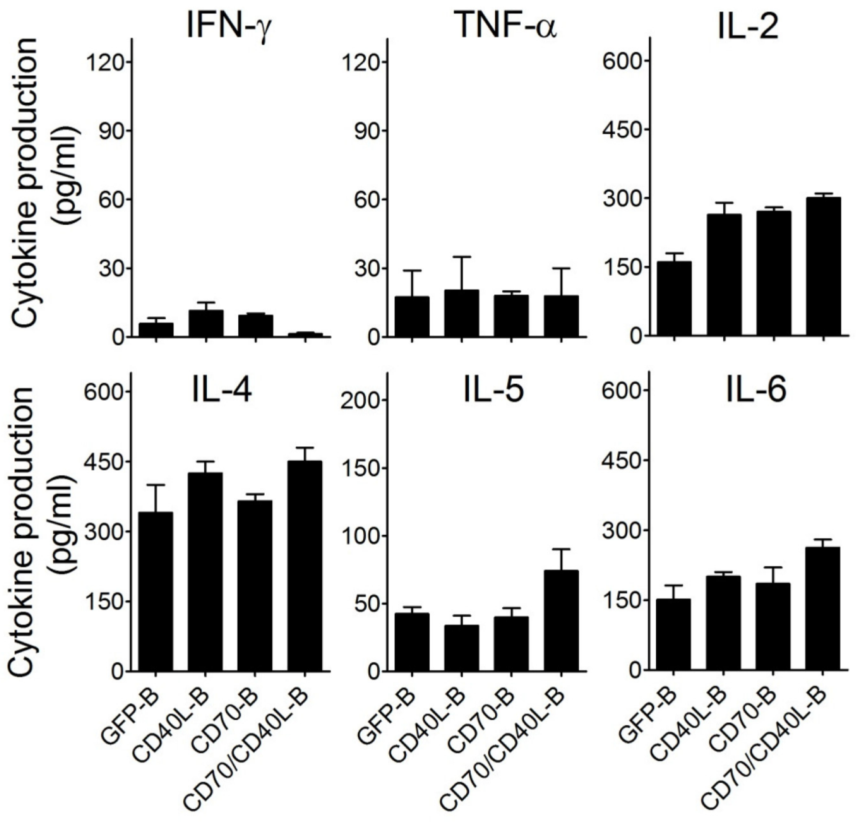


**Supplementary Figure S1: Evaluation of cytokine profiles from *ex vivo*-activated B-cells modified to express additional costimulatory ligands.** *Ex vivo*-generated B-cells were cultured *in vitro* in the presence of 10 μg/mL anti-CD40 Antibodies and 10 ng/mL IL-4. After 2 day post-transduction, the culture supernatants were measured for specific cytokine with cytometric bead-based LEGENDplex ELISA kit, according to the manufacturer's instructions. Results represent the average amount of cytokines from 2-independent experiments with SD (*bars*).

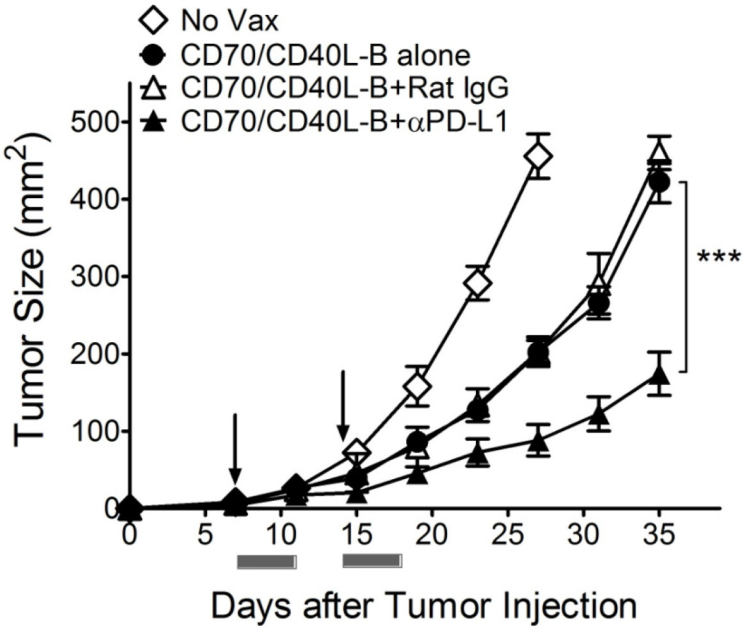


**Supplementary Figure S2: Administration of isotype control rat IgG had no effects on tumor growth.** B6 mice (4 per group) were inoculated subcutaneously on day 0 with 3 x $10^5$ B16 cells, and received intravenously with $Trp2_{180}$-loaded CD70/CD40L-B-cells on day 7, and 14 (*vertical arrow*). Anti-PD-L1 and normal rat IgG were administered as described in *Materials and Methods*. Non-vaccinated mice (No Vax) and rat IgG-non-treated mice (CD70/CD40L-B-cells alone) were included as controls. Tumor sizes were determined in individual mice by measurements of two opposing diameters and are presented as tumor areas in $mm^2$. *Gray bars*, time period of PD-1 blockade. *Points*, mean for each group of mice; *bars*, SD. *P* values were calculated using 2-way ANOVA test (***$P < 0.001$).

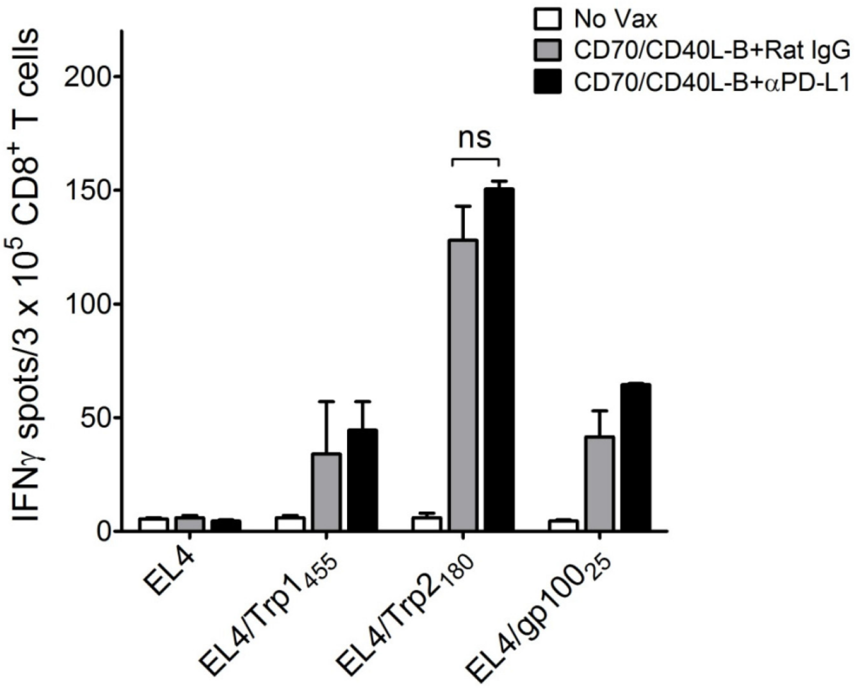


**Supplementary Figure S3: Administration of *ex vivo*-activated B-cells expressing CD40L and CD70 induces concurrent CD8 T cell responses capable of recognizing numerous antigens.** In a parallel experiment with Figure 6C, splenocytes from tumor-bearing mice from the two vaccinated groups were evaluated for antigen-specific CD8 T-cells by antigen-induced IFN-γ production against peptide-pulsed EL4 (EL4/$Trp1_{455}$, EL4/$Trp2_{180}$, and EL4/$gp100_{25}$, respectively) and un-pulsed EL4 cells (negative control). $Trp1_{455}$, TAPDNLGYA; $Trp2_{180}$, SVYDEFVWL; $gp100_{25}$, EGSRNQDWL. Results represent the average number of spots from triplicate wells with SD (*bars*) of the means. *P* values were calculated using 1-way ANOVA test (ns, not significant).

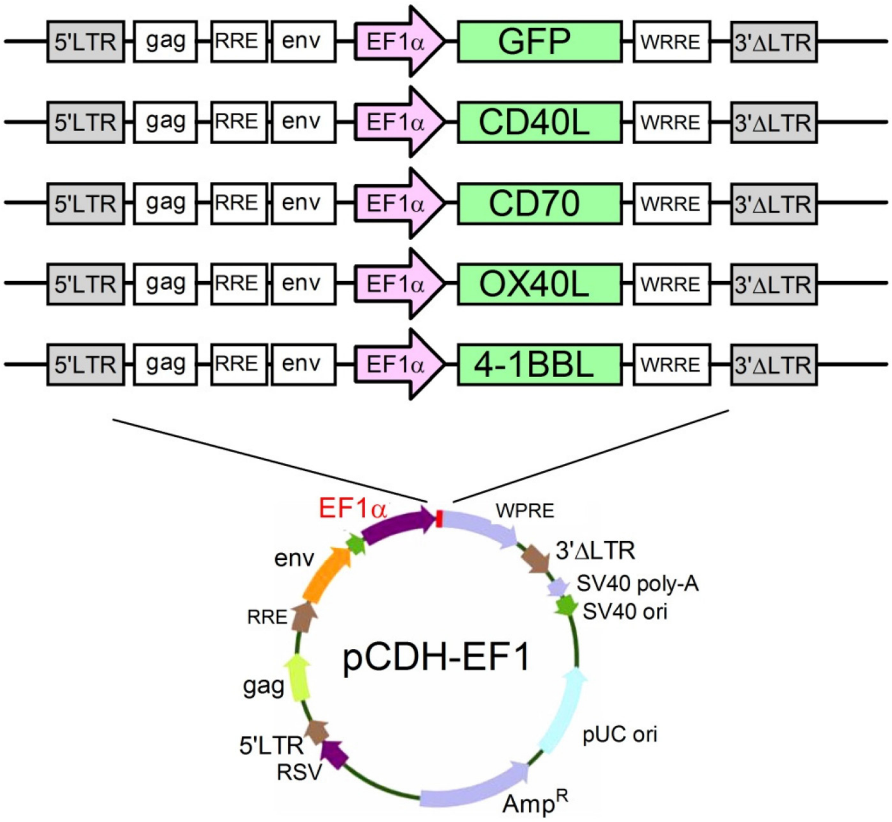


**Supplementary Figure S4: Schematic diagram of the constructs with costimulatory ligands mouse CD40L, CD70, OX40L, and 4-1BBL.** The cDNA encoding the interest genes are derived from mature DCs by extracting RNA using with *BspE*I and *Sal*I restriction site hanging at the 5' and 3' terminal respectively. The amplified genes are then inserted into the pCDH-EF1 lentiviral vector that has a constitutive elongation factor 1α (EF1α) promoter for transcription of cloned cDNA insert. Green fluorescence protein (GFP) was used as control. *RRE*, Rev response element; *WPRE*, Woodchuck hepatitis virus posttranscriptional regulatory element.

**Supplementary Table S1: Primer sequences for cloning of co-stimulatory ligands**

| Gene | Primers |
|---|---|
| CD40L | Forward : 5’-TCCCGGCCCTTCCGGAATGATAGAAACATACAGCCA-3’<br>Reverse : 5’-GAGGTTGATTGTCGACTCAGAGTTTGAGTAAGCCAA-3’ |
| CD70 | Forward : 5′-TCCCGGCCCTTCCGGAATGATTCCGGAGGAAGGTCG-3’<br>Reverse : 5’-GAGGTTGATTGTCGACTCAAGGGCATATCCACTGAA-3’ |
| OX40L | Forward : 5’-TCCCGGCCCTTCCGGAATGGAAGGGGAAGGGGTTCA-3’<br>Reverse : 5’-GAGGTTGATTGTCGACTCACAGTGGTACTTGGTTCA-3’ |
| 4-1BBL | Forward : 5’-TCCCGGCCCTTCCGGAGCCACCATGGACCAGCACACACTTGATGTG-3’<br>Reverse : 5’-GAGGTTGATTGTCGACCTACTATCATTCCCATGGGTTGTCGG-3’ |

**Supplementary Table S2: Primer sequences for detection of anti-apoptotic molecules**

| Gene | Primers |
|---|---|
| BCL-2 | Forward : 5′- GCTACCGTCGTGACTTCGCAGA-3′<br>Reverse : 5′-GTGTGCAGATGCCGGTTCAGGT-3′ |
| Bcl-xL | Forward : 5′- CGGAGAGCGTTCAGTGATCTAA-3′<br>Reverse : 5′- GATAGGTGGCCATCCAACTTGC-3′ |
| Bax | Forward : 5′- CCACCAGCTCTGAACAGATCAT-3′<br>Reverse : 5′- TCCACGTCAGCAATCATCCTCT-3′ |
| GAPDH | Forward : 5′- CAACTATTGGTGCTGGCACAAC-3′<br>Reverse : 5′- CTCGATCTTACAGCACAAGCCA-3′ |
